# Type 1 diabetes and anxiety in adolescence. A case report

**DOI:** 10.1192/j.eurpsy.2022.1106

**Published:** 2022-09-01

**Authors:** A. Franco Soler, A. Cerame, P. Coucheiro Limeres

**Affiliations:** Hospital Universitario José Germain, Psychiatry Department, Leganés, Spain

**Keywords:** adolescence, Type 1 diabetes, Anxiety, adjustment disorder

## Abstract

**Introduction:**

Type 1 diabetes (T1D) incidence is increasing around the world, being the third chronic medical condition in childhood. It is characterized by pancreatic β-cell loss which leads to insulin deficiency. Treatment includes insulin medication and lifestyle changes. Youngsters with T1D are at a high risk of psychological comorbidity (depression, anxiety, eating disorders), and especially anxiety symptoms have been correlated with worse diabetes control.

**Objectives:**

Our purpose is to examine the impact of T1D in adolescence regarding a case report and literature review.

**Methods:**

We present the case of a 14-year-old female with T1D onset and no psychiatric history. She is referred to our service three months after the T1D onset. Both her parents and the patient were anxious about the diagnostic implications and the treatment. The patient sometimes refused to follow insulin treatment and was angry and labile. Both family and individual assessment interviews were accomplished.

**Results:**

Regarding our assessment and after coordination with endocrine service (doctor and nurse) we diagnosed an [F43.23] Adjustment Disorder (acute, with mixed anxiety and depressed mood). Following recent evidence Acceptance and Commitment Therapy and Family Therapy were the election treatment with good outcomes. The patient was released after 3 months of follow-up.

**Conclusions:**

T1D treatment entails lifestyle changes and self-control, which may be stressful and challenging for adolescents and their families, causing mental health problems. Since learning self-care and emotional coping strategies can improve both psychological well-being and glucose management, an interdisciplinary approach including psychological care, especially on the onset, can be crucial.

**Disclosure:**

No significant relationships.

